# Tumour cell invasiveness and response to chemotherapeutics in adipocyte invested 3D engineered anisotropic collagen scaffolds

**DOI:** 10.1038/s41598-018-30107-3

**Published:** 2018-08-23

**Authors:** Robert D. Hume, Sara Pensa, Elizabeth J. Brown, Peter A. Kreuzaler, Jessica Hitchcock, Anke Husmann, Jonathan J. Campbell, Annabel O. Lloyd-Thomas, Ruth E. Cameron, Christine J. Watson

**Affiliations:** 1 Department of Pathology, University of Cambridge, Tennis Court Road, Cambridge, CB2 1QP UK; 2 Department of Pharmacology, University of Cambridge, Tennis Court Road, Cambridge, CB2 1QP UK; 3 Department of Biochemistry, University of Cambridge, Tennis Court Road, Cambridge, CB2 1QP UK; 4 Department of Materials Science and Metallurgy, 27 Charles Babbage Road, Cambridge, CB3 0FS UK

## Abstract

Breast cancers are highly heterogeneous and their metastatic potential and response to therapeutic drugs is difficult to predict. A tool that could accurately gauge tumour invasiveness and drug response would provide a valuable addition to the oncologist’s arsenal. We have developed a 3-dimensional (3D) culture model that recapitulates the stromal environment of breast cancers by generating anisotropic (directional) collagen scaffolds seeded with adipocytes and culturing tumour fragments therein. Analysis of tumour cell invasion in the presence of various therapeutic drugs, by immunofluorescence microscopy coupled with an optical clearing technique, demonstrated the utility of this approach in determining both the rate and capacity of tumour cells to migrate through the stroma while shedding light also on the mode of migration. Furthermore, the response of different murine mammary tumour types to chemotherapeutic drugs could be readily quantified.

## Introduction

Breast cancer mortality is a consequence of tumour metastasis to a variety of sites including lung, brain and bone. Distinguishing tumours that will metastasize from those that will not is challenging and often results in un-necessary or inappropriate treatment of women with primary breast cancer. As a step towards personalised medicine, it is essential to be able to predict the capacity of a tumour to metastasize and to respond to particular therapeutic regimes. A further confounding factor is the heterogeneous nature of many breast tumours where a subclone of tumour cells may behave differently to the bulk tumour. Thus, we sought to develop an *in vitro* culture model that accurately recapitulates the breast stroma in 3D and allows individual cells from a tumour biopsy fragment to invade this stromal milieu. In addition, we aimed to develop techniques that permit assessment/visualization of this ‘metastatic’ potential and the response of invading cells to a panel of therapeutic drugs.

A variety of 3D *in vitro* culture models have been generated for studies of both the normal and malignant breast epithelium, all of which have defined utility^1–13^. The majority of these consist of cells traversing an isotropic (non-directional) lattice. However, directional migration of tumour cells has been shown to be strongly influenced by chemical gradients and/or directional cues provided by the organisational structure of the scaffolding molecules that cells adhere to, known as the extracellular matrix (ECM). For example, the ECM protein collagen is frequently aligned in an anisotropic (directional) manner in breast tumours with poor prognosis^14–18^. Therefore it is essential to recapitulate this collagen-rich anisotropic ECM structure *in vitro* in any study of breast cancer cell migration.

Another crucial component of the tumour stroma is the fat pad, which provides an adipocyte-rich environment that the breast tumour cells must traverse/negotiate. Adipocytes are responsive to various hormones and secrete a variety of components including adipokines that influence migration^19,20^. Thus, it is important to incorporate this integral stromal component into any 3D model.

In previous work, we developed 3D anisotropic engineered collagen scaffolds and demonstrated their value as a tool to measure the ability of individual cells from established breast cancer cell lines to invade the scaffold^21^. However, breast tumours are heterogeneous in nature, and metastases arise from a minor yet critical subclone(s) of tumour cells that evolve within a specific tumour microenvironment. In this study, we sought to develop our model further and to utilise it to investigate the capacity of cells from primary tumours to migrate into a surrounding stroma. This is more relevant for breast tumour growth and metastasis *in vivo* as the invasive capacity of cells is analysed in the context of intact tumour architecture. Furthermore, this preserves the immediate tumour microenvironment comprising cancer-associated fibroblasts, immune cells, cytokines and ECM.

Since there are multiple sub-types of breast cancer, and individual breast cancers are highly heterogeneous, we sought to compare the invasive behaviour of tumour cells derived from mouse mammary tumour models where carcinogenesis is initiated by different oncogenes. The first tumour model analysed was the well-established MMTV-*Wnt1* transgenic mouse model where overexpression of the *Wnt1* proto-oncogene is driven by the MMTV promoter, resulting in adenocarcinoma development in FVB mice^22^. The second tumour model utilised was the TUBO cell line, derived from a mammary carcinoma that developed in a Balb/c-Her2/neu transgenic mouse, and injected into a syngeneic mouse mammary gland^23^. This model was chosen as overexpression of HER2 occurs in approximately 25% of human breast cancers and is related to a poorer prognosis than the more common oestrogen receptor positive disease^24^. Another advantage is that TUBO tumours allow faster experimental turnaround, as they arise approximately 5 weeks after cell inoculation. Once established, primary tumours were harvested and frozen for subsequent experiments to provide a biobank of near-identical tumour biopsies.

To further develop our *in vitro* model into a cancer therapeutic testing tool, a selection of available drugs were screened as a proof of principle. For this assessment, we selected three inhibitors of different pathways implicated in a variety of migratory mechanisms and processes. Firstly, we chose the Rho-associated protein kinase (ROCK) inhibitor, Y-27632 (denoted ROCKi hereafter), which affects a wide range of processes including proliferation, apoptosis, cell migration, adhesion, oncogenic transformation and the cytoskeleton^25,26^. Secondly, the pan-matrix metalloproteinase (MMP) inhibitor, GM6001, was selected as ECM remodelling and subsequent cancer cell migration is linked to MMP expression^27,28^. Lastly, the pan-ErbB inhibitor Canertinib was selected due to the role of the ErbB receptors EGFR^29^, HER2^30^ and ErbB-4^31^ in breast tumour growth and progression.

Here we report significant enhancements to our original model by introducing pre-adipocytes into the scaffolds and differentiating these into lipid-filled adipocytes^32^ followed by implanting primary mammary tumour fragments and measuring both the ability of tumour cells to invade the scaffold and observing their mode of migration. Our results show that this enhanced 3D model permits the distinct migratory behaviours of cells from different types of tumours to be observed and quantitatively analysed. Furthermore, this approach provides a rapid screen of the response of invading cells to therapeutic drugs. Thus, we can provide an *in vitro* platform for drug screening that will be useful in identifying efficacy and toxicity and may be utilised for personalised breast cancer medicine.

## Results

### Engineered Tumour-Stroma Interaction Model (ET-SIM) and tumour fragment cultures

**Figure 1:**
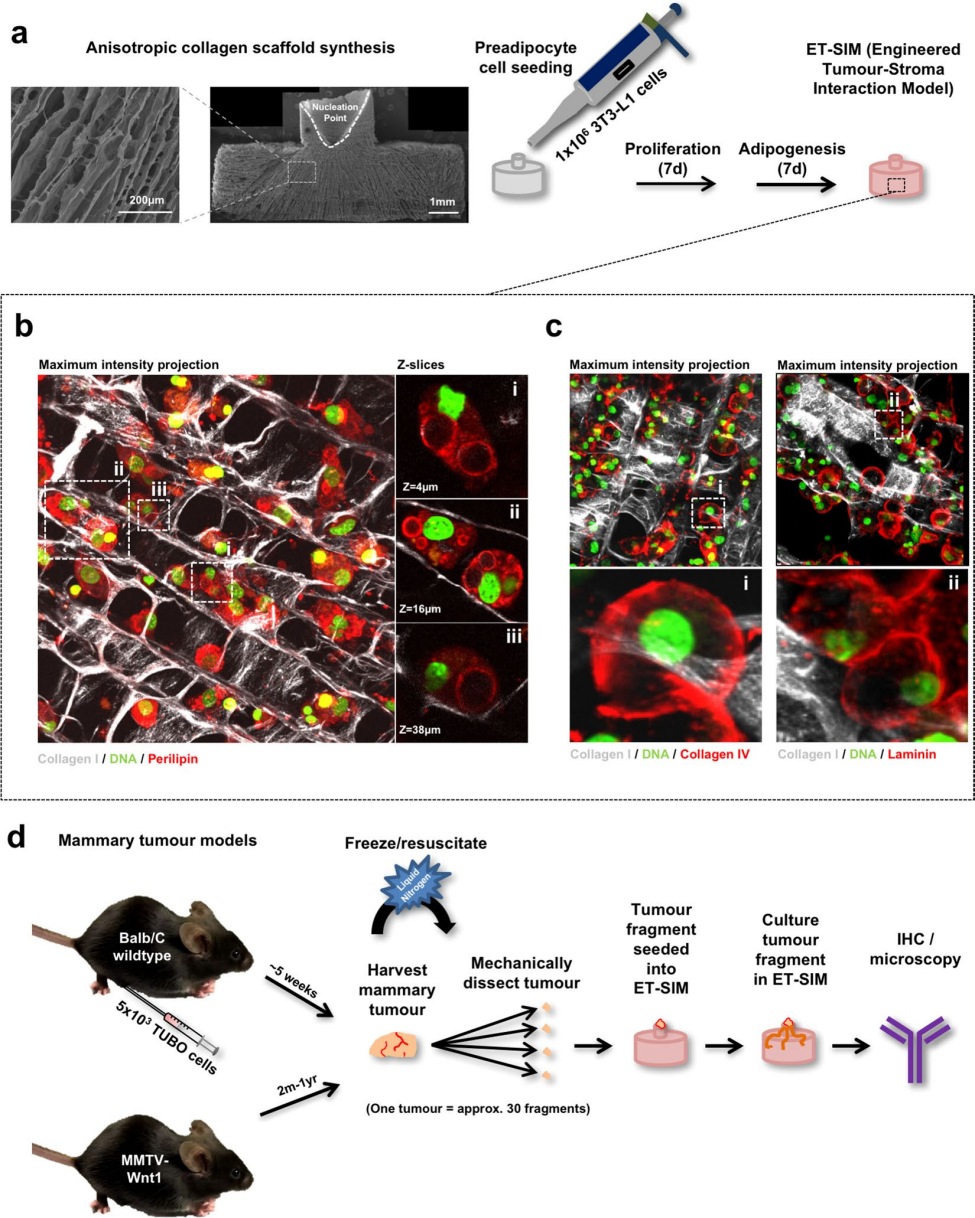
ET-SIM (Engineered Tumour-Stroma Interaction Model) and tumour fragment culture strategy. (**a**) Scanning electron micrograph (SEM) of anisotropic collagen scaffold (scaffold nucleation point marked with a white dotted line) with directional collagen pores and schematic of ET-SIM (Engineered Tumour-Stroma Interaction Model) culture. (**b**) Whole mount immunostained ET-SIM culture imaged using second harmonic generation (SHG, collagen I, grey) and two photon fluorescence (2pf) microscopy z-stacks. Nuclei are stained with green fluorescent dye SYTO16 (green). Lipids are stained with anti-perilipin (red). Z-stacks are displayed as maximum intensity projections (left) and individual magnified z-sections (i-iii, right). (**c**) Whole mount immunostained ET-SIM culture imaged using SHG and 2pf microscopy z-stacks. Nuclei are stained with green fluorescent dye SYTO16 (green). Basement membrane proteins are stained with anti-collagen IV (red, left) and anti-laminin (red, right). Z-stacks are displayed as maximum intensity projections. Magnified maximum intensity projections shown in (i) and (ii). (**d**) Schematic of tumour fragment and ET-SIM co-culture.

To recapitulate the anisotropic collagen matrix surrounding mammary tumours, we synthesised 3D anisotropic collagen scaffolds using our previously described freeze drying protocol^21^. A synthetic fat pad was generated within the scaffolds by seeding preadipocytes (3T3-L1 cells), culturing for 7 days to fill the scaffold and then switching to differentiation media to induce these cells to differentiate into mature adipocytes^32^. The work flow to generate this model, that we name Engineered Tumour-Stroma Interaction Model (ET-SIM), is illustrated in Fig. 1a. Whole scaffold immunostaining in conjunction with two-photon fluorescence microscopy (2pf) and second harmonic generation (SHG) was used to visualise the mature adipocytes and detect collagen I, respectively. Combined 3D z-stacks show mature adipocytes exhibiting intracellular immunostaining for the lipid marker perilipin, within anisotropic collagen pores (Fig. 1b).

Previously, it has been reported that 3T3-L1 cells express the basement membrane proteins collagen IV and laminin upon adipogenesis^33,34^. As shown in Fig. 1c, both proteins were deposited in a pericellular fashion. The scaffolds therefore comprise not only directional collagen I and adipocytes but also basement membrane proteins, all of which are present in the mammary tumour microenvironment. After thawing, tumours were dissected into fragments, and seeded into ET-SIM cultures as shown schematically in Fig. 1d.

### ET-SIM can distinguish tumour cell migration phenotypes

**Figure 2:**
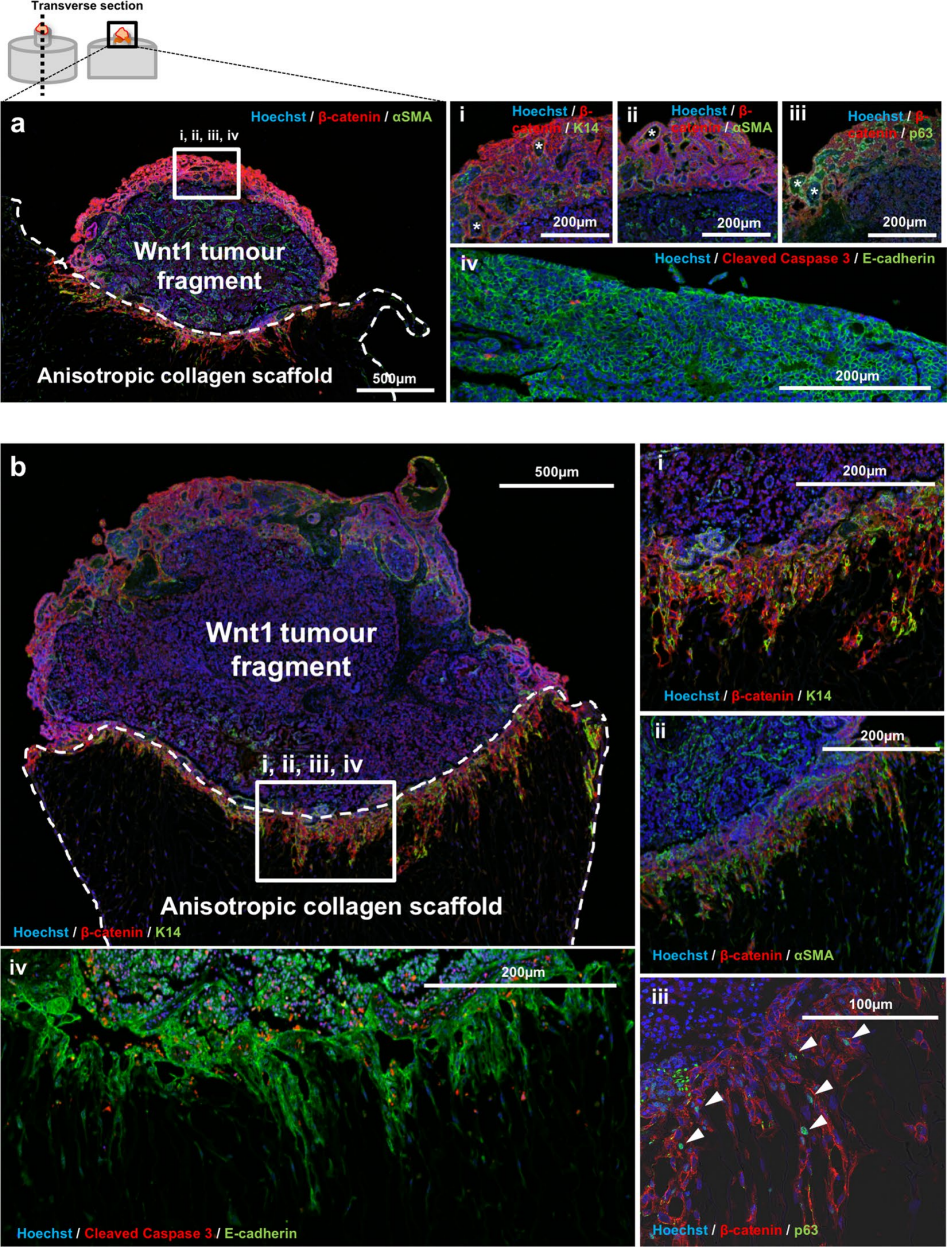
Wnt1 driven tumour fragment co-culture and tumour cell analysis. (**a**) Immunohistochemistry (IHC) of Wnt1 tumour fragment (top), seeded into the nucleation point (white dotted line) of anisotropic collagen scaffolds/ET-SIM (bottom), cultured for 72 hours, embedded in paraffin and transversely sectioned (diagram, top left). A tile scan of the area containing the tumour fragment and the top of the scaffold is shown in (a). Magnified images of the free edge of the tumour fragment (a, white box) is shown in (i-iv). DNA is marked using Hoechst (blue). Luminal tumour cells are marked with anti-β-catenin (red, i-iii) and E-cadherin (green, iv). Lumen marked as*. Basal tumour cells are marked with anti-cytokeratin-14^+^ (K14, green, i), anti-α-smooth muscle actin^+^ (αSMA, green, ii) and anti-p63 (nuclear, green, iii). Apoptotic cells are marked with anti-cleaved caspase-3 (CC3, red, iv). (**b**) Using the same markers as in (a), the edge of the tumour in contact with an anisotropic collagen scaffold (b, white box) and (i-iv), shows collective migration of K14^+^/αSMA^+^/p63^+^ basal and β-catenin^+^/E-cadherin^+^ luminal cells. Arrowheads in (iv) show p63^+^ nuclei.

Following 72 hours of Wnt1 tumour fragment culture in ET-SIM cultures, IHC revealed low levels of cleaved caspase-3 (CC3) at both the free edge of the tumour as well as the edge in contact with the scaffold indicating that the tumour cells remain viable in ET-SIM cultures (Fig. 2a,iv,b,iv). The scaffold-free edge retains the original tumour architecture showing the characteristic reverse bilayer phenotype of Wnt1 tumours where basal cells, defined by their expression of cytokeratin-14 (CK14), α-smooth muscle actin (αSMA) and nuclear p63, line the lumen^35,36^ (Fig. 2a,i-iii, lumen marked as*). Luminal cells expressing β-catenin and E-cadherin show a hyperplastic, disorganised cobblestone phenotype (Fig. 2a,i-iv). In contrast, where the tumour contacts the scaffold, both basal (CK14^+^, αSMA^+^, p63^+^) and luminal (E-cadherin^+^, β-catenin^+^) cells were seen invading collectively into the surrounding stroma (Fig. 2b,i-iv). These tumour cells migrated parallel to the pores of collagen in the scaffold, demonstrating the directional effect of anisotropic ECM architecture on cell movement (Fig. 2b,i-iv). The resultant tendril-like epithelial formations conform closely to the anisotropic porous architecture of the scaffold, indicating that these scaffolds may present 3D surfaces for a structured spatial analysis of tumour infiltration.

**Figure 3:**
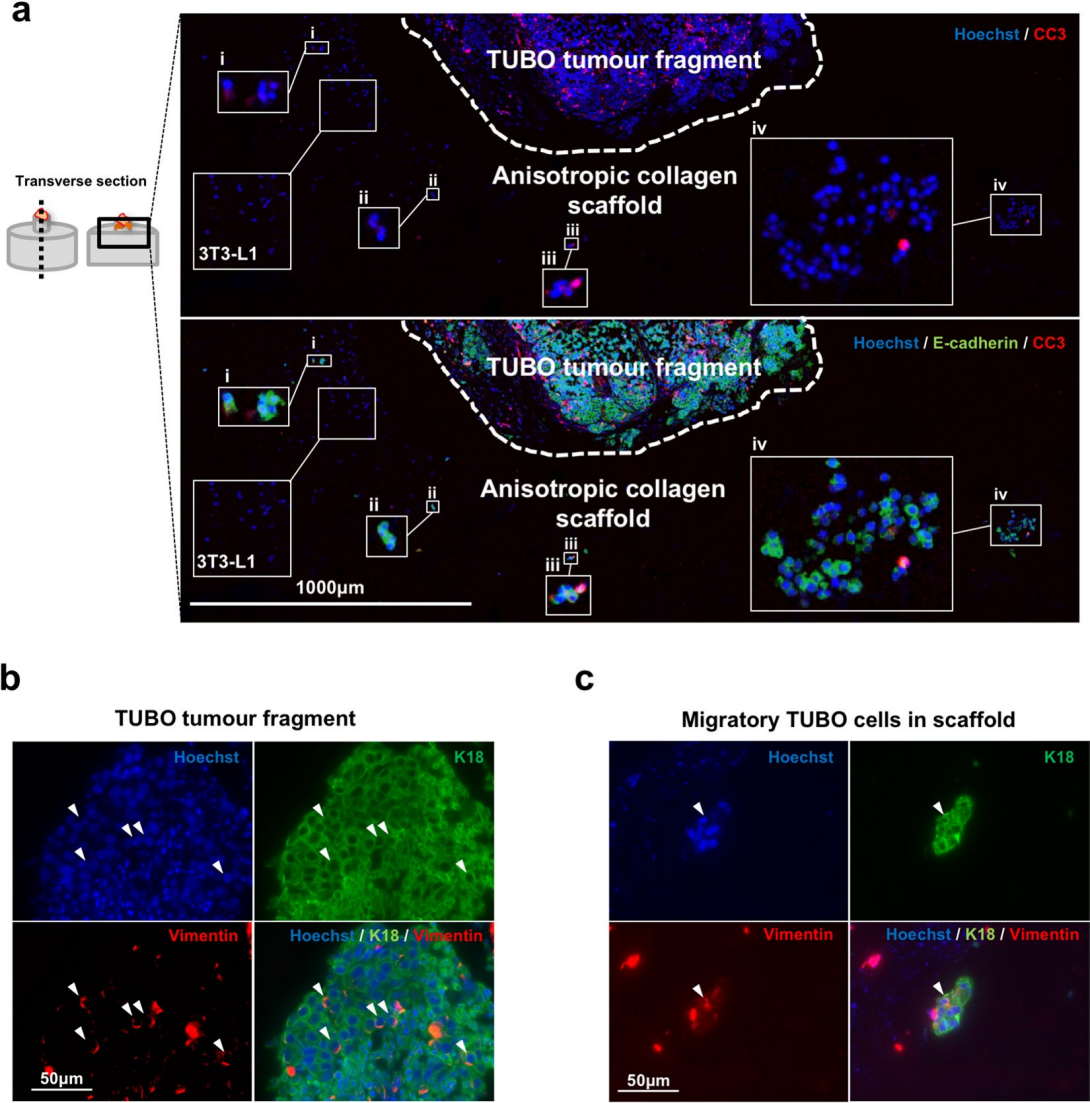
TUBO tumour fragment co-culture and tumour cell analysis in ET-SIM. (**a**) Immunohistochemistry (IHC) of TUBO (Her2-neu overexpressing) tumour fragments (top), seeded into the nucleation point (white dotted line) of anisotropic collagen scaffolds (bottom) with 3T3-L1 adipocytes (insert labelled 3T3-L1), cultured for 72 hours, embedded in paraffin and transversely sectioned (diagram, left). The area shown is a tile scan of the tumour fragment and the top of the scaffold (diagram, left). DNA is marked using Hoechst (blue). TUBO tumour cells are marked with anti-E-cadherin (green). Apoptotic cells are marked with anti-cleaved caspase-3 (CC3, red). Magnified images of migratory TUBO cells are shown in inserts (i-iv). (**b**) IHC of TUBO cells within the bulk of the seeded tumour fragment. TUBO cells are marker with anti-cytokeratin-18 (K18, green) and epithelial-to-mesenchymal (EMT) transition marker anti-vimentin (red). Stochastic expression of vimentin is localised around one edge of TUBO cell nuclei (arrowheads). (**c**) IHC of migratory TUBO tumour cells within the anisotropic collagen scaffold with similar stochastic vimentin expression in K18^+^ TUBO cells (arrowhead).

IHC observation of longitudinal sections of TUBO tumour fragment in ET-SIM cultures (Fig. 3a, schematic) show the majority of cells within the tumour fragment and migratory clusters that were CC3 negative and therefore still alive (Fig. 3a). This demonstrates that TUBO primary tumour fragments and migratory cells are also viable in ET-SIM cultures.

In stark contrast to the collective outpouring of cells from *Wnt1* tumour fragments, the majority of migratory TUBO tumour cells migrated as small clusters (<10 cell) (Fig. 3a,i-iii) or rarer large clusters (>50 cells) separate from the seeded tumour fragment (Fig. 3a,iv). Migratory cells were also observed at a range of distances from the nucleation point (Supplementary Fig. 1). Stochastic expression of the epithelial-to-mesenchymal (EMT) marker vimentin was observed around one edge of cell nuclei in both the seeded tumour fragment and in cells that had migrated into the scaffold (Fig. 3b,c). Thus through the use of two different tumour models we have demonstrated that distinct migratory phenotypes can be readily assessed in ET-SIM cultures.

### ET-SIM as a breast cancer therapeutic testing platform – 72 hours migration study

Following on from this validation of ET-SIM as a method to distinguish different modes of tumour cell migration, we proceeded to test ET-SIM as a superior cancer therapeutic testing platform. MMTV-*Wnt1* tumour fragments were cultured with and without DMSO (vehicle controls, ROCKi, GM6001 and Canertinib inhibitors for 72 hours in either ET-SIM cultures (i.e. co-cultured with differentiated 3T3-L1 adipocyte cells) or empty scaffolds to elucidate both therapeutic efficacy and adipocyte influence on migration. Migration distance was quantified through analysis of IHC sections of fixed samples using a modification of our previously published method^21^.

**Figure 4:**
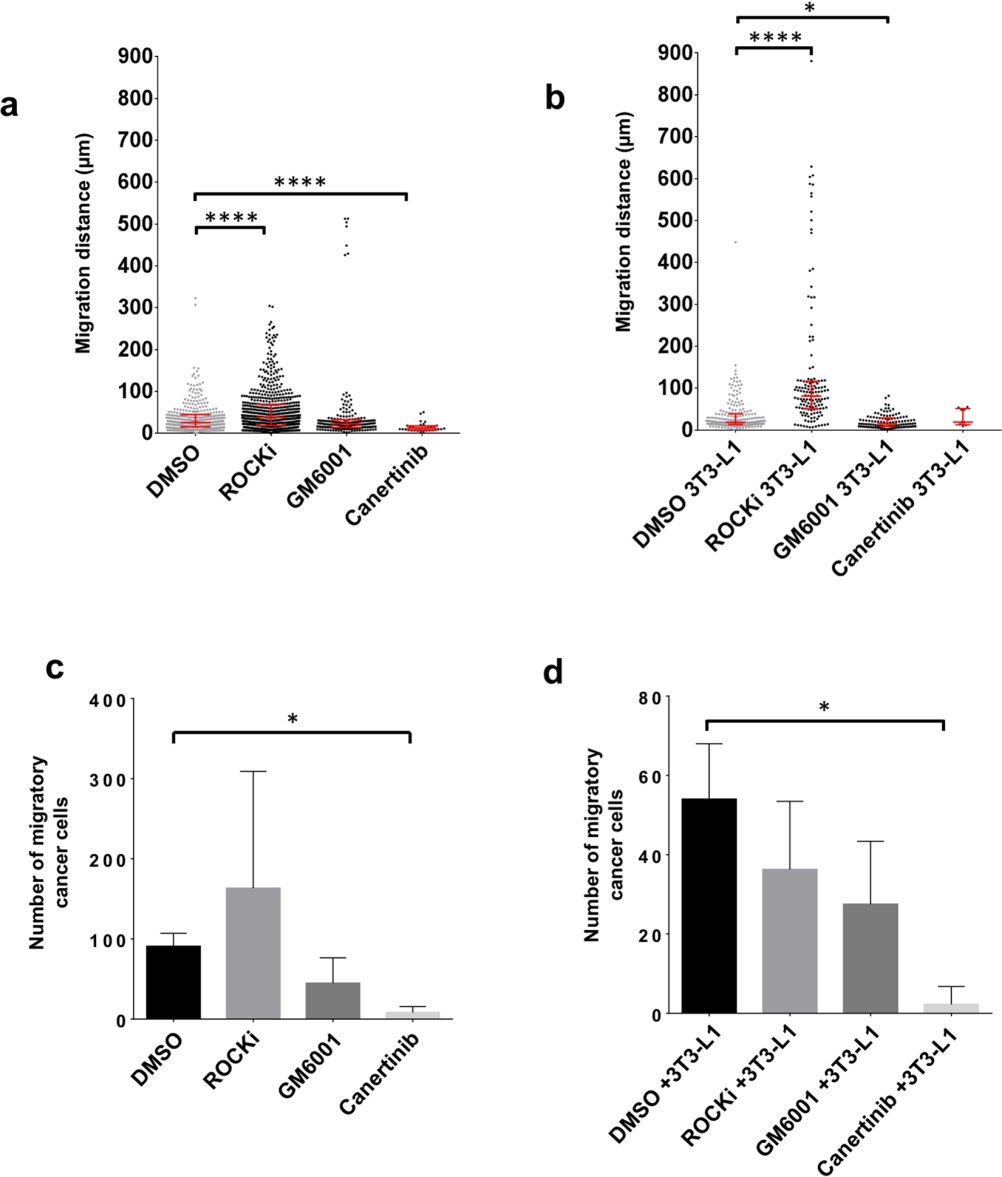
Analysis of tumour cell migration and response to pathway inhibitors (72 hours). Comparison of Wnt1 tumour cell migratory distance, from the anisotropic collagen scaffold nucleation point to within the scaffold, after 72 hours culture in the absence (**a**) or presence (**b**) of differentiated 3T3-L1 cells (known as ET-SIM), with/without pathway inhibitors ROCKi, GM6001 and Canertinib, all statistically compared to DMSO (vehicle control). The total number of migratory Wnt1 tumour cells within the scaffolds, in the absence (**c**) or presence (**d**) of differentiated 3T3-L1 cells (known as ET-SIM), with/without the pathway inhibitors ROCKi, GM6001 and Canertinib compared to DMSO (vehicle control). The following statistical analyses were applied: non-parametric unpaired/matching Kruskal-Wallis ANOVA with a Geisser-greenhouse correction combined with a Dunn’s multiple comparison test, *p < 0.05, ****p < 0.0001. Sample size n = 4.

After 72 hours, treatment of tumour fragments in empty scaffolds with Canertinib resulted in a reduced migration distance, when compared to vehicle (DMSO) controls (Fig. 4a). This could have resulted from high levels of cell death before the cells were able to migrate into the scaffold (Supplementary Fig. 2). In contrast, treatment of tumour fragments in empty scaffolds with ROCKi resulted in enhanced migration compared to DMSO vehicle controls (Fig. 4a). Similarly, ROCKi treatment of ET-SIM cultures resulted in an enhanced cell migration distance (Fig. 4b). Furthermore, after 72 hours, treatment of tumour fragments in ET-SIM cultures with GM6001 resulted in reduced migration, when compared to vehicle (DMSO) controls (Fig. 4b).

In addition to the migration distance of each cell, we measured the total number of cells that had migrated from the tumour fragment into the scaffold. After 72 hours, the total number of migratory cells was significantly decreased by treatment with Canertinib, when tumour fragments were seeded in either empty scaffolds (Fig. 4c) or ET-SIM cultures (Fig. 4d).

Each treatment condition was also compared individually for tumour fragments seeded in empty scaffolds versus ET-SIM cultures, at the 72 hour time point, to investigate the influence of adipocytes. During vehicle (DMSO) treatment, cells in ET-SIM cultures exhibited decreased migration distance and a diminished number of migratory cells compared to empty scaffolds (Supplementary Fig. 3a,e). Interestingly, for ROCKi treatment, the opposite effect was observed in ET-SIM cultures with increased migration distance compared to empty scaffolds although the number of migratory cells was decreased by the adipocytes (Supplementary Figs 3b,f, 4). This suggests that adipocytes facilitate the migration of certain subsets of tumour cells while suppressing the invasiveness of others. Thus ET-SIM cultures have the capacity to reveal the heterogeneity of tumours and allow even small numbers of invasive cells to be detected. Furthermore, the response of such invasive cells to therapeutic drugs can be readily assessed and the most appropriate drug treatment to suppress the migration and/or survival of the subpopulations of metastatic tumour cells determined. We conclude that adipocytes have a complex modulatory effect on the outcome of drug treatment and these data highlight the value and potential importance of using an adipocyte invested 3D environment within a drug testing platform.

### ET-SIM as a breast cancer therapeutic testing platform – 10 days migration study

**Figure 5:**
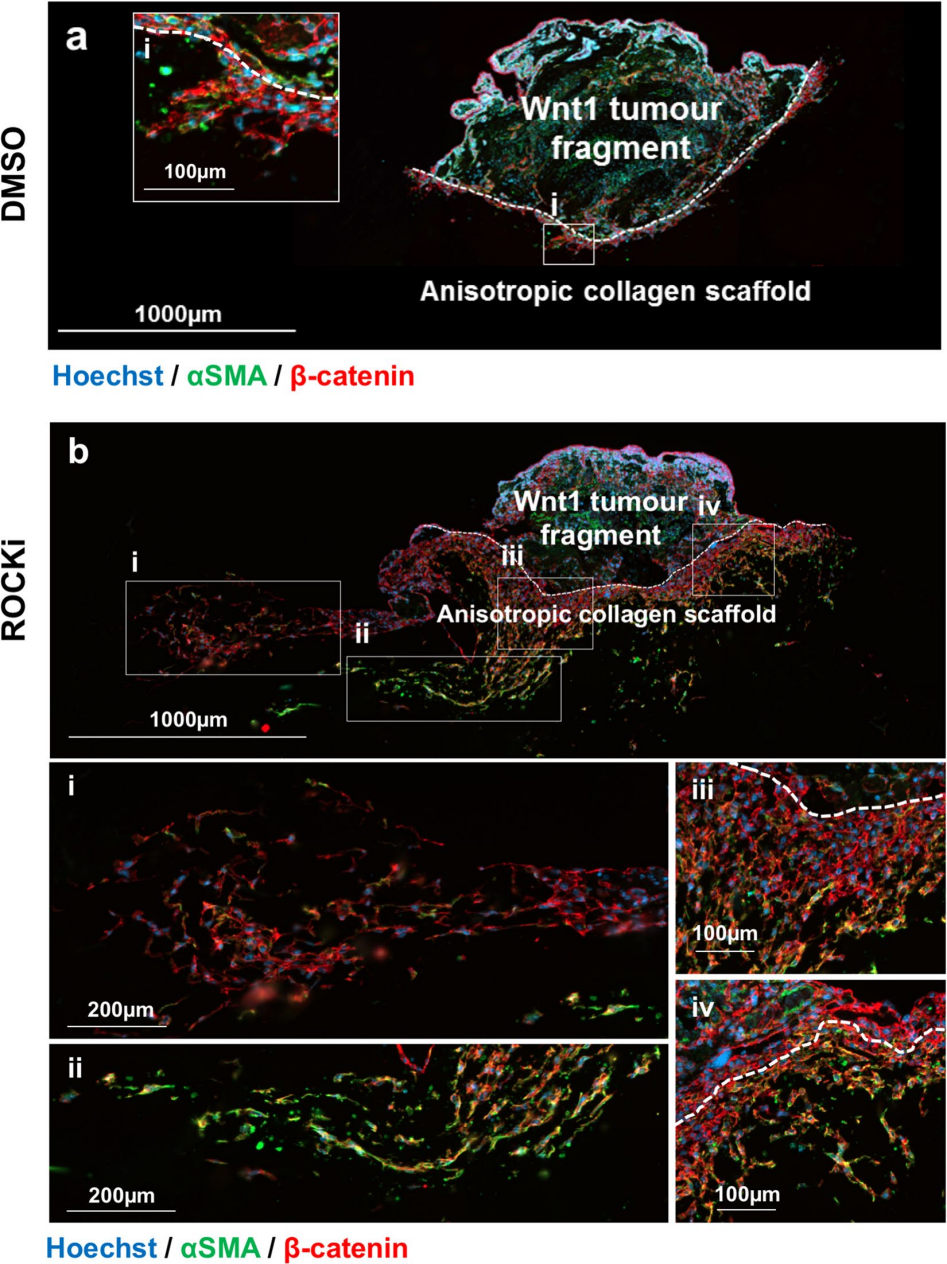
Wnt1 tumour cells treated with ROCKi exhibit increased migration. Immunohistochemistry analysis of Wnt1 tumour fragment seeded into an empty anisotropic collagen scaffold nucleation point (white dotted line), cultured for 10 days then embedded in paraffin and transversely sectioned. Migratory cancer cells are marked with αSMA (green) and β-catenin (red) and DNA is marked with Hoechst. Panel (**a**) is in the presence of DMSO (vehicle control) for 10 days and (i) shows migratory cells at <500 µm near the nucleation point. Panel (**b**) is in the presence of ROCKi (Y-27632) for 10 days. (i) and (ii) show migratory cells at distances at >500 µm. (iii) and (iv) show migratory cells at <500 µm near the nucleation point.

To investigate whether the responses observed at 72 hours were restricted to the initiation of migration only, we carried out a longer time course culturing tumour fragments for up to 10 days. IHC observations revealed that the extent of tumour cell migration was strikingly increased in ROCKi samples compared to vehicle controls in empty scaffolds (Fig. 5). Tracts of cells can be seen protruding in all directions from the tumour fragment, with some cells migrating more than 20 times the distance of vehicle controls. The most distal cells (>500 µm) from the tumour fragment showed a thin mesenchymal phenotype and expressed both β-catenin and αSMA (Fig. 5b,i,ii). Cells found closer to the tumour fragment tended to be more clustered (Fig. 5b,iii,iv).

**Figure 6:**
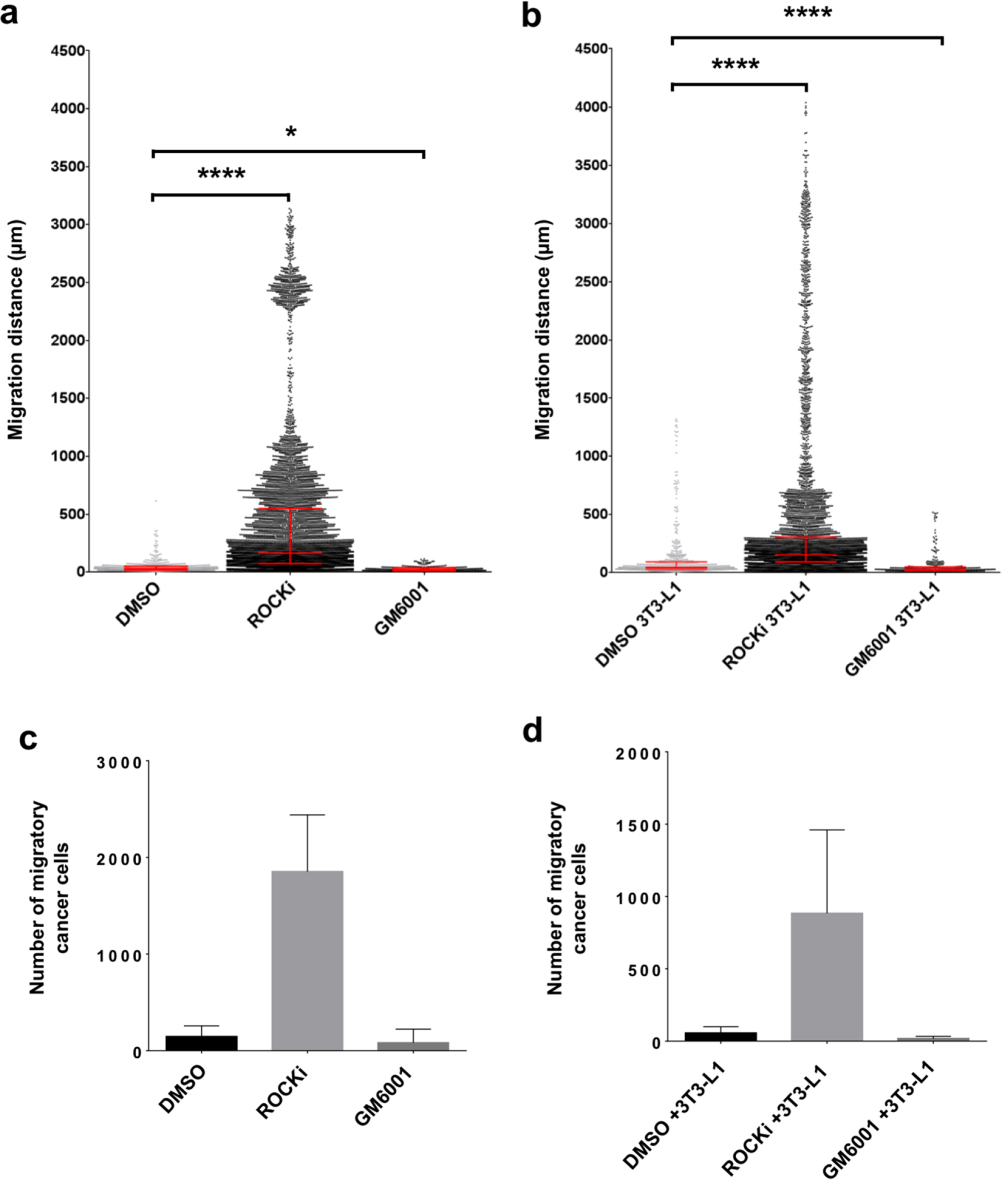
Analysis of tumour cell migration and response to pathway inhibitors (10 days). Comparison of Wnt1 tumour cell migratory distance, from the anisotropic collagen scaffold nucleation point to within the scaffold, after 72 hours culture in the absence (**a**) or presence (**b**) of differentiated 3T3-L1 cells (known as ET-SIM), with/without pathway inhibitors ROCKi, GM6001 and Canertinib, all statistically compared to DMSO (vehicle control). The total number of migratory Wnt1 tumour cells within the scaffolds, in the absence (**c**) or presence (**d**) of differentiated 3T3-L1 cells (known as ET-SIM), with/without the pathway inhibitors ROCKi, GM6001 and Canertinib compared to DMSO (vehicle control). The following statistical analyses were applied: non-parametric unpaired/matching Kruskal-Wallis ANOVA with a Geisser-greenhouse correction combined with a Dunn’s multiple comparison test, *p < 0.05, ****p < 0.0001. Sample size n = 4.

After 10 days, Canertinib completely abolished cell migration in both empty scaffolds and ET-SIM cultures with no migratory cells found in any scaffolds, demonstrating the high efficacy of Canertinib at longer time-points regardless of adipocyte status (data not shown). The MMP inhibitor, GM6001, showed anti-migratory effects in both empty scaffold and ET-SIM cultures, supporting the concept that MMP induced matrix remodelling assists tumour cell migration^28^ (Fig. 6a,b). ROCKi effects were clearly more pronounced in longer term treatments, with a subset of cells reaching >3000 µm in empty scaffolds and >4000 µm in ET-SIM cultures (Fig. 6a,b). Indeed, all treatments at 10 days showed an increase in migration distance when comparing each treatment individually for empty scaffolds versus ET-SIM (Supplementary Fig. 5). This suggests that adipocytes promote later stages of cell migration in all therapeutic regimes tested at 10 days underscoring the need for oncologists and drug discovery laboratories to be conscious of their stromal influence when selecting medicines.

Lastly, the total number of cancer cells that migrated at 10 days was analysed. Although not significant, these data suggest ROCKi treatment increases the total number of migratory cancer cells independent of adipocyte status when compared to DMSO vehicle controls (Fig. 6c,d). This is suggestive of a possible pro-survival/proliferative effect of ROCKi on migratory cells in Wnt1 tumours. In concordance with 72 hour data, adipocyte co-culture showed a decreasing trend in the total number of migratory cancer cells in all samples (Supplementary Fig. 5). Interestingly, at 10 days there is therefore an inverse trend of migration distance (Supplementary Fig. 5a–d) and number of migratory cancer cells (Supplementary Fig. 5d–g) after 10 days when adipocytes are added to tumour cultures. This indicates that adipocyte pro-migratory effects are not on the entire population of tumour cells but a subset. It also may suggest adipocytes to be suppressive of certain cellular subsets, preventing them from gaining a migratory phenotype.

### Optical clearing allows visualisation of tumour/ET-SIM cultures to analyse therapeutic effect

**Figure 7:**
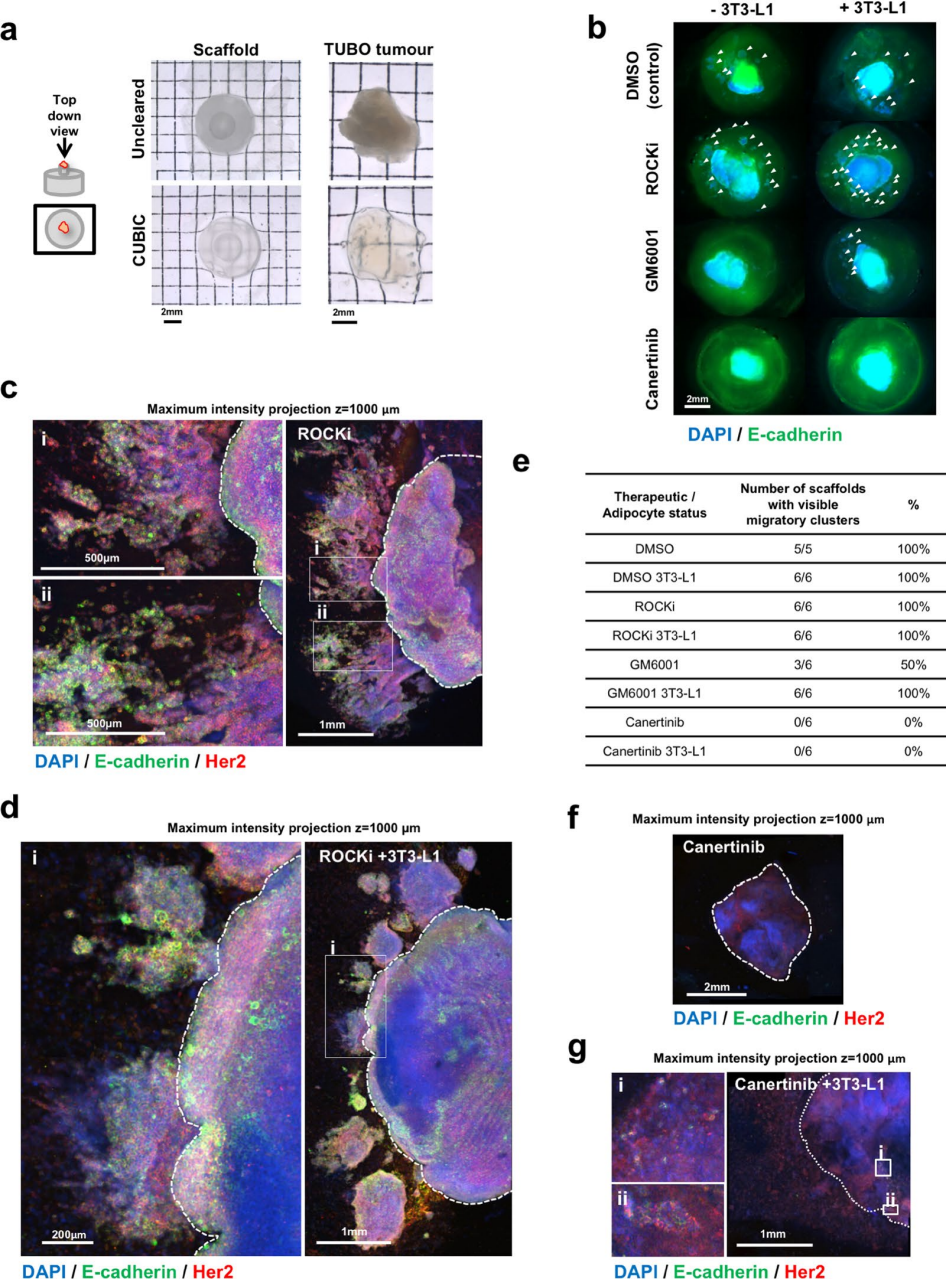
Optical clearing of TUBO tumours in anisotropic collagen scaffolds and cancer therapeutic testing. (**a**) Transmission stereoscopic images of uncleared and optically cleared (CUBIC) anisotropic collagen scaffold and TUBO (Her2-neu overexpressing) tumour from a top down view. (**b**) Fluorescent stereoscopic images of TUBO tumour fragments in scaffolds, cultured for 10 days with/without adipocytes (3T3-L1), treated with DMSO (control), ROCKi (Y-27632), GM6001 or Canertinib, CUBIC cleared and immunostained for E-cadherin (green) and Her2 (not shown). Cell nuclei are stained with DAPI (blue). Clusters of cancer cells that have migrated away from the central tumour fragment are marked with arrowheads. (**c**,**d**) Large tile scan z-stack (1 mm depth) confocal microscopy images of ROCKi treated TUBO tumour fragments in anisotropic collagen scaffolds without/with adipocytes (3T3-L1), cleared and stained as described in (**b**) with DAPI (blue), E-cadherin (green) and Her2 (red). (i) and (ii) show magnified images of migratory clusters of Her2 and E-cadherin positive cells. (**e**) Quantification of the number of tumour/scaffolds that contain one or more visible migratory cell clusters. (**f**,**g**) Large tile scan z-stack (1 mm depth) confocal microscopy images of Canertinib treated TUBO tumour fragments in anisotropic collagen scaffolds without/with adipocytes (3T3-L1), cleared and stained as described in (**b**) with DAPI (blue), E-cadherin (green) and Her2 (red). (i) and (ii) show few non-migratory E-cadherin and Her2 cells seen in the seeded tumour fragment. No migratory cells were observed in the scaffold with Canertinib treatment at this magnification.

CUBIC optical clearing methods^37^, which increase tissue transparency by matching refractive indices, permit 3D immunolocalisation of protein and allows deeper laser penetration and z-sectioning by confocal microscopy of whole mounts. Our lab has shown TUBO tumours can be optically cleared and imaged using this approach^38^. Therefore we adapted this method to analyse scaffolds, tumours and their combination and were able to image these with stereoscopic imaging (Fig. 7a,b), 2pf/SHG microscopy (Supplementary Fig. 6b) and confocal microscopy (Fig. 7c–g). Clearing successfully increased transparency (Fig. 7a) and permitted deep confocal laser z-penetration of 1000 µm (Fig. 7c–g). Furthermore, it allowed 3D visualisation of tumour cells and anisotropic collagen ECM in TUBO and Wnt1 tumour fragments (Supplementary Fig. 6b).

After 10 days of culture with therapeutics, cleared TUBO tumour cultures revealed colonies of cells that had migrated away from the tumour fragment in all control and ROCKi samples regardless of adipocyte status (Fig. 7b,e). In contrast, GM6001 effectively suppressed colony migration in 50% of samples when seeded in empty scaffolds, indicating an anti-migratory effect of MMP inhibition (Fig. 7b,e). This effect was reversed when the tumours were embedded in ET-SIM cultures (Fig. 7b,e) demonstrating that adipocytes exert a pro-migratory effect during GM6001 treatment. Similar to its effect on Wnt1 tumours, Canertinib showed a pronounced anti-migratory affect in TUBO tumours with no migratory colonies visible in 100% of samples regardless of adipocyte status (Fig. 7b,e–g). Confocal microscope tile scan z-stacks revealed that cancer cells in ROCKi treated tumours migrated xy-distances >1000 µm (Fig. 7c,i,ii) from the seeded tumour fragment (dotted line, Fig. 7c,d) and displayed heterogeneous expression of Her2 and E-cadherin. These techniques underscore the superiority of the ET-SIM system in combination with tissue clearing and 3D imaging to analyse tumour migration and drug efficacy.

## Discussion

Current cell culture models are limited in their ability to recapitulate the cellular and ECM complexity of the tumour microenvironment. Thus, there is much interest in building 3D culture systems that incorporate stromal components that accurately reflect the *in vivo* environment^5^. We have pioneered an organotypic *in vitro* assay that recapitulates essential aspects of the *in vivo* mammary tumour microenvironment. The ET-SIM system improves upon current models by combining anisotropic 3D spatial ECM architecture, which has been shown to support cancer cell migration^21^, with an adipocyte rich environment. Here we show that ET-SIM supports culture of tumour fragments, permits visualization and quantitative analysis of distinct modes of tumour cell migration, reveals tumour heterogeneity, and can be used to test tumour cell responsiveness to drug treatments. We suggest that these features demonstrate potential utility as a drug discovery platform.

Reducing the burden of animal models for experimental purposes has both moral and financial incentives. Through the mechanical dissection of mammary tumours into multiple fragments and seeding into separate scaffold cultures, ET-SIM allowed multiple experimental conditions to be tested following the sacrifice of each single mouse. This study therefore, reduced the number of animals required for this study, one of the gold standards set out by the “3Rs” principles (Refinement, Reduction and Replacement)^39^. This could have major implications for much larger commercial drug studies if our model were to be adopted.

Previous reports have shown breast tumours can be grouped according to three different tumour-associated collagen signatures (TACS)^14^. Invasive MMTV-*Wnt1* tumours remodel surrounding stromal ECM into a TACS-3 phenotype where collagen fibres run perpendicular to the tumour boundary creating localised anisotropy that provides a highway for metastatic cells. Importantly, a similar TACS-3 phenotype has been reported in human breast tumours that are associated with poor patient prognosis^15^. Our system mimics this *in vivo* microenvironment by seeding mammary tumour fragments into anisotropic collagen scaffolds. While other studies have reproduced collagen anisotropy *in vitro* using biaxial collagen gels^11,13,40–43^, our method uses a freeze drying approach^21^ to create collagen pores aligned perpendicular to the surface of a nucleation point in all directions. As the tumour fragments are seeded at the nucleation point, the scaffold mimics an anisotropic ECM at the periphery of the tumour in all directions. Additionally, these collagen scaffolds are not subject to the shrinkage issues associated with hydrogels mediated by cellular compaction^44^.

To study adipocyte influences on tumour cell migration, we co-cultured mammary tumour fragments arising from either Wnt1 or Her2 overexpressing cells within ET-SIM cultures. In all culture conditions it was clear that the presence of adipocytes increased the migration distance of tumour cells after 10 days of culture. Adipocytes have been implicated in breast cancer progression through paracrine and endocrine signalling and can increase breast cancer cell line invasion *in vitro*^10,19,45^. Interestingly, at 72 hours adipocytes had an anti-migratory effect in some conditions suggesting that adipocytes may secrete factors at earlier time points that inhibit specific drug activities. Indeed, consistent with this notion, exposing breast cancer cells to adipocyte conditioned medium has been shown to reduce the therapeutic efficacy of trastuzumab^46^. Collectively these observations highlight the need to incorporate adipocytes into assays of drug efficacy.

Despite adipocytes positively influencing the distance that Wnt1 tumour cells migrate after 10 days in culture, adipocytes showed a tendency to reduce the overall number of migratory cells suggesting that they act as a physical selective barrier or filter – impeding some cells while facilitating penetration of more aggressive cancer cells. Importantly, ET-SIM thus reveals heterogeneous cellular behaviour and is therefore ideally suited to identify whether drugs target cellular subpopulations responsible for metastatic spread rather than generically de-bulking a tumour without effectively eliminating it.

While examining the specific effects of three different inhibitors on Wnt1 tumour fragments we observed a striking increase in Wnt1 tumour cell migration during ROCKi treatment. Previous literature is divided on the effects of ROCKi with some studies showing that ROCK inhibition supresses or inhibits cancer cell migration in pre-aligned collagen gels^41,42^ while others show that ROCK inhibition increases invasive potential^47–49^, suggesting that ROCKi exerts cell type specific and context dependent effects. Of note, ROCKi has been shown to promote cell survival and colony forming efficiency when used in organoid cultures of human breast, mouse mammary gland and MMTV-*Wnt1* tumours^50–53^. Enhanced cell survival would explain the increased number of migratory cells but not necessarily the increased migration distance observed in ROCKi treated samples. Collectively, these data indicate that caution should be used when considering ROCK inhibitors as therapeutic agents. Migration distance was decreased although not entirely prevented, by the MMP inhibitor GM6001 suggesting that migration in our ET-SIM system is, at least partly, reliant on ECM remodelling and MMP function. Canertinib however, completely abrogated both TUBO and Wnt1 tumour cell migration in terms of both distance and number of cells after 10 days of treatment showing it to be the most effective therapy for these tumours.

A limitation of our 3D cultures is the possibility that both inhibitory and stimulatory factors did not reach the core of the tumour fragment, due to the inefficient diffusion of nutrients in our static system. Tumour cells touching the scaffold may also be subject to diffusion issues, as they must obtain nutrients through the full scaffold thickness. Higher levels of cleaved caspase 3, a marker of apoptosis and some other forms of programmed cell death, were observed in this population, as compared to tumour cells at the free edge, and may have been a consequence of restricted diffusion. Nutrient restriction could also induce autophagy, although we have not investigated this directly. It is worth noting that the anisotropic structure of the scaffold should permit increased diffusion to the nucleation point. This is where the tumour fragments were seeded and where tumour cells touching the scaffold were located. Directional pores orientated from the edge of the scaffold - a nutrient rich area - may permit diffusion of essential nutrients to this location. Further investigation is required into the diffusion characteristics of the scaffold itself.

Optical clearing techniques have vastly enhanced the depth of imaging achievable with confocal and 2pf microscopy. We have demonstrated that anisotropic collagen scaffolds, ET-SIM cultures, tumour fragments and their combination can be cleared using CUBIC successfully to enable fluorescent stereoscopic imaging and analysis of tumour cell migration. Additionally, CUBIC enabled deep (1000 µm) z-sectioning using immunofluorescence coupled with confocal microscopy, which facilitated a more detailed investigation into migratory cell phenotype and location. This simple technique provided novel insights into different forms of tumour cell migration. Furthermore, it allowed quantitative analysis of the diverse effects of pathway-specific inhibitory drugs and has significant potential as a tool for rapid ‘first pass’ investigation of drug efficacy.

In summary, we have pioneered a new enhanced organotypic mammary tumour culture system. We demonstrate that this system provides both qualitative and quantitative insight into mammary tumour cell migration, the complex bivalent effects of adipocytes on this process and the effects of anti-metastatic drugs. We envisage that humanisation of this model will pave the way for improved personalised medicine strategies for patients with breast cancer. Patient derived tumour biopsy fragments containing both stromal and tumours cells, derived from different geographical regions and cultured in our ET-SIM system, would provide oncologists with another tool to use in concert with standard strategies to provide personalised medicine for breast cancers.

## Methods

### Animal models

Syngeneic TUBO mammary tumours were established by implanting 5 × 10^3^ TUBO cells into the abdominal mammary gland of BALB/c females. This cloned cell line was established from a mammary carcinoma that spontaneously arose in a BALB-neuT mouse and carries the Her-2/neu oncogene driven by the MMTV promoter^23,54^. Tumours were harvested before exceeding humane end points (4–5 weeks) and frozen for future use. MMTV-*Wnt1* mice were crossed onto an FVB background for quicker tumour onset. Tumours were harvested at humane end points (2-12 months).

### Tumour freezing

Tumours were placed in cryovials, covered with freezing medium (DMEM/F12 GlutaMAX (Gibco, 41965–039), 40% FBS (Gibco, 10500064), 6% DMSO), placed immediately into a freezing container (ThermoFisher) followed by −80 °C overnight incubation. Cryovials were then stored in liquid nitrogen until required. For resuscitation cryovials were from hand warmed. Once thawing was first observed, room temperature medium was added. Tumours were decanted into tubes containing fresh tumour medium (DMEM/F12, 10% FCS, 1x Penicillin/Streptomycin (Invitrogen, 15140122), 5 μg/ml Insulin (Sigma, 11882), 10 ng/ml Epidermal Growth Factor (Sigma, E4127)) making sure not to carry across any freezing medium. Decanting was repeated to wash away any remaining freezing medium. Tumours were mechanically fragmented using a scalpel into 1–3 mm^3^ pieces for tumour fragment seeding.

### Anisotropic collagen scaffold synthesis

Anisotropic collagen scaffolds were synthesized using our freeze drying method^21^. Briefly, collagen from bovine Achilles tendon (Sigma Aldrich, UK) was added to 0.05 M acetic acid overnight, homogenised and aspirated into engineered molds containing a copper pin. Using a freeze drier ice was then sublimated and the scaffolds cross-linked with 70% ethanol +33 mM 1-ethyl-3-(3-dimethylaminopropyl)-carbodiimide hydrochloride +6 mM *N*-hydroxysuccinimide and sterilized in 70% ethanol overnight. Scaffolds were washed in phosphate buffered saline (PBS) and incubated in maintenance medium (MM) (DMEM (Gibco, 41965), 10% heat inactivated foetal bovine serum (FBS) (Gibco, 10500064) and 1x penicillin/streptomycin (Gibco, 15140–122)) overnight to assure sterility.

### Engineered Tumour-Stroma Interaction Model (ET-SIM)

3T3-L1 cells were cultured in MM between passages 4–20. 3T3-L1 cells were seeded and differentiated in anisotropic collagen scaffolds using a modified previously published method^32^. Briefly, 1 × 10^6^ 3T3-L1 cells were seeded into scaffolds (1 ml medium, 24 well plate), left to proliferate for 7 days and differentiated using an adipogenic cocktail (MM supplemented with 1 μg/ml insulin, 0.25 μM dexamethasone (Sigma) and 3-isobutyl-1-methylxanthine (IBMX, Sigma)) for 7 days, changing media every other day. At this point scaffolds were moved into 6 well plates ready for tumour fragment seeding. All cell cultures were in 5% CO_2_ at 37 °C in a humid incubator.

### Tumour fragment seeding and ET-SIM culture

Mechanically fragmented tumour pieces were seeded using sterile forceps into anisotropic collagen scaffold nucleation points with/without previously differentiated 3T3-L1 cells in 6 well plates. 1 ml of tumour media was carefully added so as not to dislodge the tumour fragment and left for 4 hours to attach. Following this an additional 4 ml of tumour media was added covering both the scaffold and tumour. A fresh 5 ml of tumour media was replaced each day and scaffolds were removed at different time points, fixed in 4% paraformaldehyde (PFA) and either paraffin embedded for immunohistochemistry or the CUBIC whole mount immunostaining protocol (see methods) was applied.

### Therapeutic testing

After 24 hours of tumour fragment culture as mentioned above the media was replaced with 5 ml of tumour media supplemented with either 35.2 µM DMSO (Sigma Aldrich, D8418), 10 µM Y-27632 (ROCKi) (Cell Guidance Systems, SM02-10), 10 µM GM6001 (Merck Millipore, 364205) or 10 µM Canertinib (Selleckchem, S1019). Media was replaced with fresh 5 ml tumour media plus therapeutic every 24 hours until fixation endpoint in 4% PFA.

### Immunohistochemistry

Paraffin embedded sections were deparaffinised, rehydrated in an ethanol series, permeabilised in 0.5% Triton-X/PBS and boiled in 10 mM sodium citrate for 11 mins in a pressure cooker for antigen retrieval. Samples were blocked in blocking buffer (10% normal goat serum (Sigma, G9023) in PBS-0.05% Triton-X (VWR, 28817.295). Primary antibodies were diluted in blocking buffer as follows: rabbit anti-β-catenin 1:1000 (Cell Signalling, 957 S), mouse anti-αSMA 1:100 (Abcam, ab7817), mouse anti-E-cadherin 1:500 (BD biosciences, 610182), mouse anti-Cytokeratin-18 pre-diluted (Progen Biotech 65028), rabbit anti-vimentin 1:100 (Cell signalling, 5741), rabbit anti-Cytokeratin-14 (Abcam, ab53115), mouse anti-p63 1:100 (Abcam, ab735), rabbit anti-cleaved caspase-3 1:800 (Cell signalling, 9664) and the nuclear dye Hoechst 33342 at 5 µg/ml (Thermofisher, H3570). The following secondary antibodies were all purchased from life technologies and were all diluted in blocking buffer 1:500: AlexaFluor 488 goat anti-rabbit (A11008), AlexaFluor 647 goat anti-rabbit (A21245), AlexaFluor 488 goat anti-mouse (A11001), and AlexaFluor 647 goat anti-mouse (A21237).

### CUBIC optical clearing and whole mount immunostaining

CUBIC clearing was based on the modified version of the protocol by Susaki *et al*.^37^, that utilizes the CUBIC R1a method (unpublished, protocol available at http://cubic.riken.jp/) as previously described by Lloyd-Lewis *et al*.^38^. Briefly, fixed samples were placed in R1a for 3 days in a dry incubator at 37 °C, changing to fresh R1a each day. Samples were blocked overnight in blocking buffer (10% normal goat serum in PBS-0.5% Triton-X). Primary antibodies were diluted in blocking buffer for 4 days at 4 °C in the following concentrations: rabbit anti-perilipin 1:50 (Cell Signalling, D418), rabbit anti-collagen IV 1:100 (Abcam, ab6586), rabbit anti-laminin 1:100 (Abcam, ab11575), rabbit anti-HER2 1:100 (Dako, A0485) and the nuclear dye SYTO16 at 1 µM (Life technologies, S7578). Secondary antibody Alexa Fluor 647 goat anti-rabbit (Life Technologies, A21245) was diluted 1:500 and incubated for 2 days at 4 °C.

### 2-photon fluorescence (2pf) and second harmonic generation (SHG) microscopy

2pf and SHG was carried out on the LaVision BioTec TriM Scope II upright 2-photon scanning fluorescence microscope using a 25x water dipping objective. Filters used were: 645–695 nm (detector 1, Alexa Fluor 647), 495–560 nm (detector 2, SYTO16) and 395–445 nm (detector 3, SHG). A fixed 1040 nm laser excited Alexa Fluor 647 and a tuneable laser at 836 nm for SYTO16 and SHG excitation.

### Confocal Microscopy

Confocal imaging was carried out on the Leica TCS SP8 inverted confocal microscope and analysed on Fiji^55^.

### Epi-fluorescence microscopy

Standard fluorescence microscopy and tile scans were carried out on the Zeiss Imager H&E and fluorescence microscope, converted into TIFFs and analysed on Fiji^55^.

### Migration analysis

Cancer cell migration was analysed using a modified version of our previously published method^21^. Briefly, using tile scans generated from IHC, cell nuclei of migratory cells were marked using Fiji and their coordinates measured. The scaffold nucleation point was segmented and saved as coordinates. The closest distance from the nucleation point for each migratory nucleus was measured using formulae in Microsoft Excel and these results were then combined in GraphPad. Cancer cell nuclei were distinguished from stromal cells using the markers αSMA and β-catenin and were defined as migratory when found in or on any part of the scaffold away from the tumour fragment.

### Statistical analyses

Migration distances with multiple samples (Figs 6a,b and 7a,b) were analysed using the non-parametric unpaired/matching Kruskal-Wallis ANOVA with a Geisser-greenhouse correction combined with a Dunn’s multiple comparison test. Migration distances comparing with/without 3T3-L1 cells (Figs 6c–f and 7c–e) were compared using the non-parametric unpaired Mann-Whitney test using a confidence level of 95%. The number of migratory cells with multiple samples (Figs 6g,h and 7f,g) was compared using an ordinary one-way ANOVA. The number of migratory cells with/without 3T3-L1 cells (Fig. 6i) was compared using an unpaired t-test. All statistical analyses were carried out using the software GraphPad Prism 6.

### Ethical approval

Work was carried out under UK Home Office Licence authority following ethical review of protocols by the local animal ethics committee of the University of Cambridge. All animal experimentation was carried out in accordance with the Animal (Scientific Procedures) Act 1986, and the European Union Directive 86/609. Mice were humanely sacrificed by dislocation of the neck.

### Data availability

The datasets generated during and/or analysed during the current study are available from the corresponding author on reasonable request.

## Electronic supplementary material

Supplementary Figures 1-6
