# Supplementary material for: Tumour cell invasiveness and response to chemotherapeutics in adipocyte invested 3D engineered anisotropic collagen scaffolds
Source: Sci Rep. 2018 Aug 23;8:12658. doi: 10.1038/s41598-018-30107-3 (PMC6107500; doi:10.1038/s41598-018-30107-3)
Supplement: Supplementary file 1 — Supplementary Figures 1-6 [file 41598_2018_30107_MOESM1_ESM.pdf]

Tumour cell invasiveness and response to chemotherapeutics in adipocyte invested 3D engineered anisotropic collagen scaffolds

Robert D. Hume[1], Sara Pensa[2], Elizabeth J. Brown[1], Peter A. Kreuzaler[3], Jessica Hitchcock[1], Anke Husmann[4], Jonathan J. Campbell[4], Annabel O. Lloyd-Thomas[1], Ruth E. Cameron[4] and Christine J. Watson[1*]

Departments of Pathology[1], Pharmacology[2] and Biochemistry[3] University of Cambridge, Tennis Court Road, Cambridge, CB2 1QP, UK and Materials Science and Metallurgy[4], 27 Charles Babbage Road, Cambridge, CB3 0FS, UK

Supplementary Figures

Supplementary Figure 1

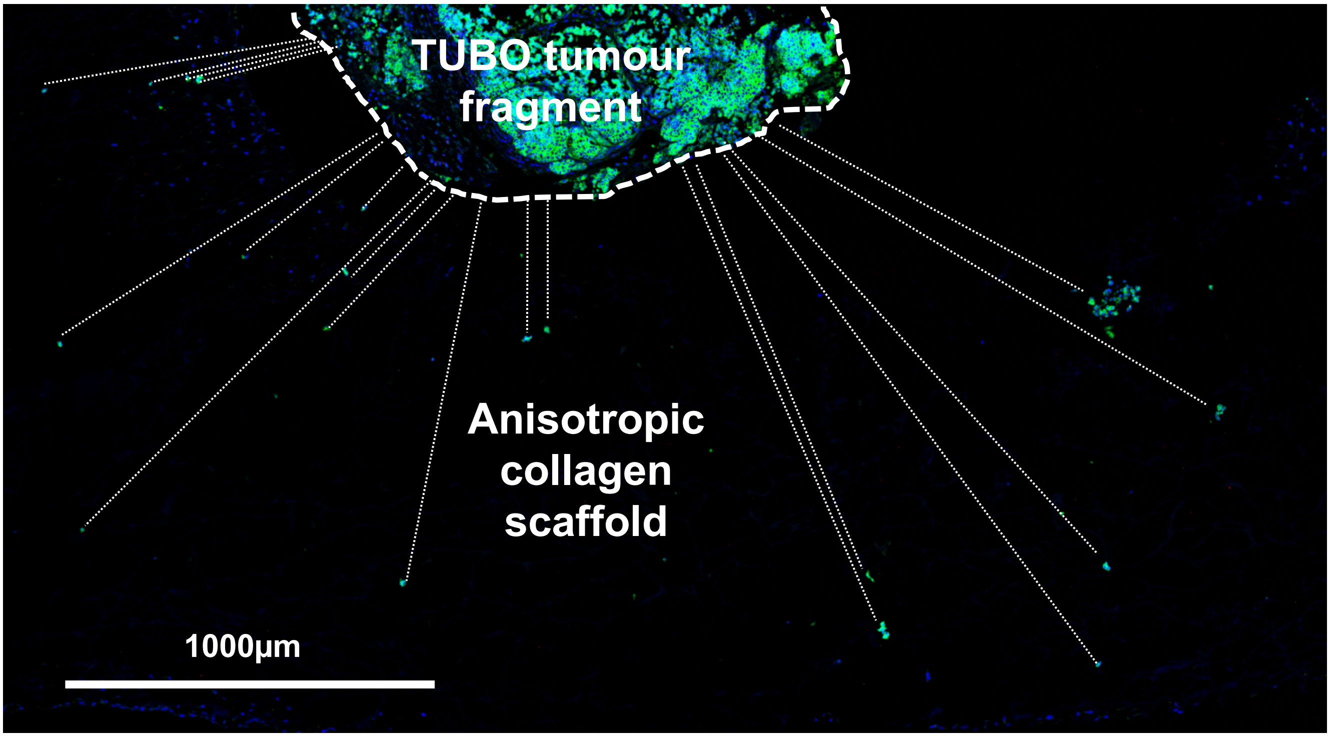

DAPI / E-cadherin

Supplementary Figure 1. TUBO tumour cells migrate to a range of distances in anisotropic collagen scaffolds. Immunohistochemistry (IHC) of TUBO (Her2-neu overexpressing) tumour fragments (top), seeded into the nucleation point (white dotted line) of anisotropic collagen scaffolds (bottom) with 3T3-L1 adipocytes, cultured for 72 hours, embedded in paraffin and transversely sectioned. DNA is marked using Hoechst (blue). TUBO tumour cells are marked with anti-E-cadherin (green). Migratory TUBO tumour cells at a range of migratory distances are marked with white straight dotted lines from the nucleation point.

Supplementary Figure 2

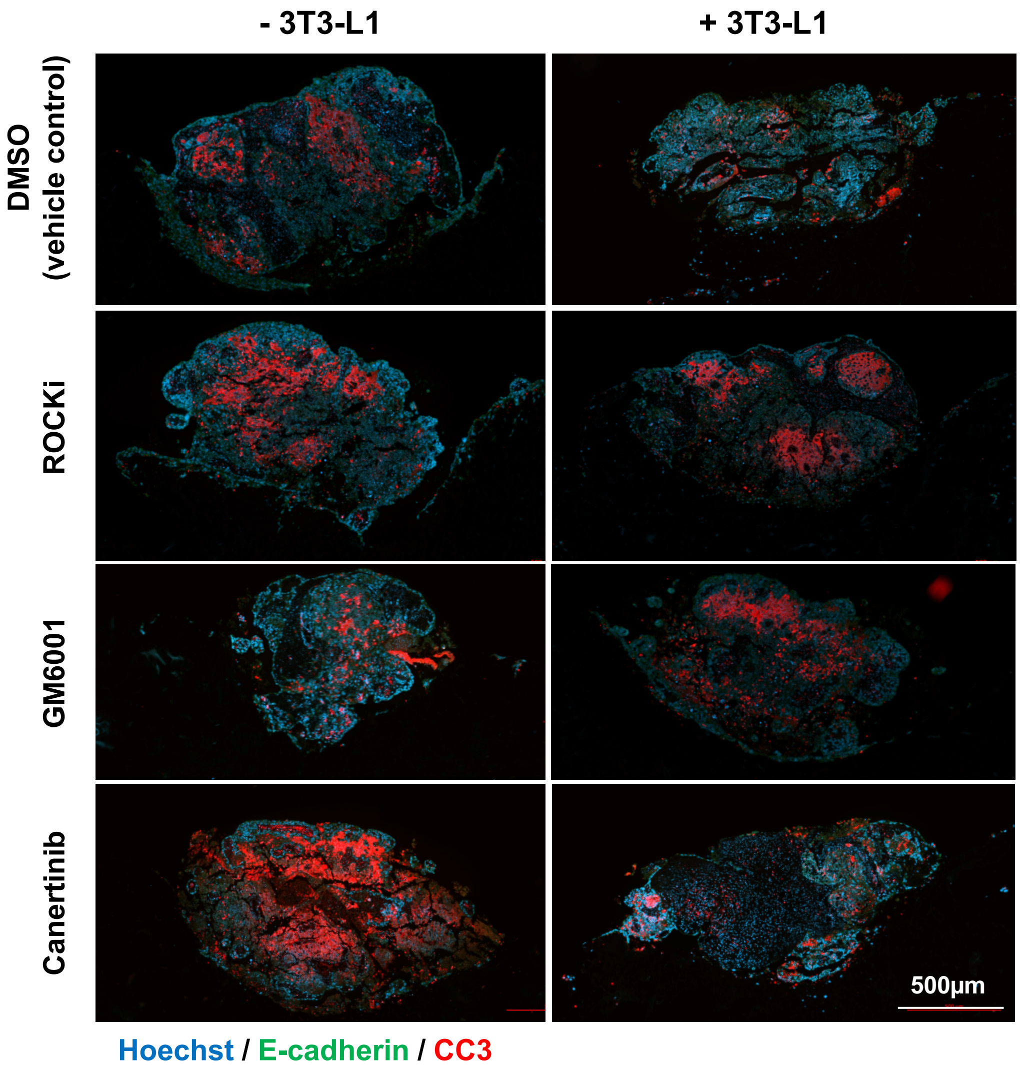

Supplementary Figure 2. Apoptosis of Wnt1 tumour fragments in anisotropic collagen scaffold in response to various cancer therapeutics. Immunohistochemical analysis of Wnt1 tumour fragments in scaffolds, cultured for 72 hours with/without adipocytes (3T3-L1), treated with DMSO (control), ROCKi (Y-27632), GM6001 or Canertinib, and immunostained with apoptosis marker anti-cleaved caspase 3 (CC3, red) and tumour cell marker E-cadherin (green). Cell nuclei are stained with Hoechst (blue). 12 random subjects were shown IHC of the different therapeutic testing strategies (n=3) in a blinded test. 100% selected Canertinib (no 3T3-L1) as showing the highest CC3 levels.

Supplementary Figure 3

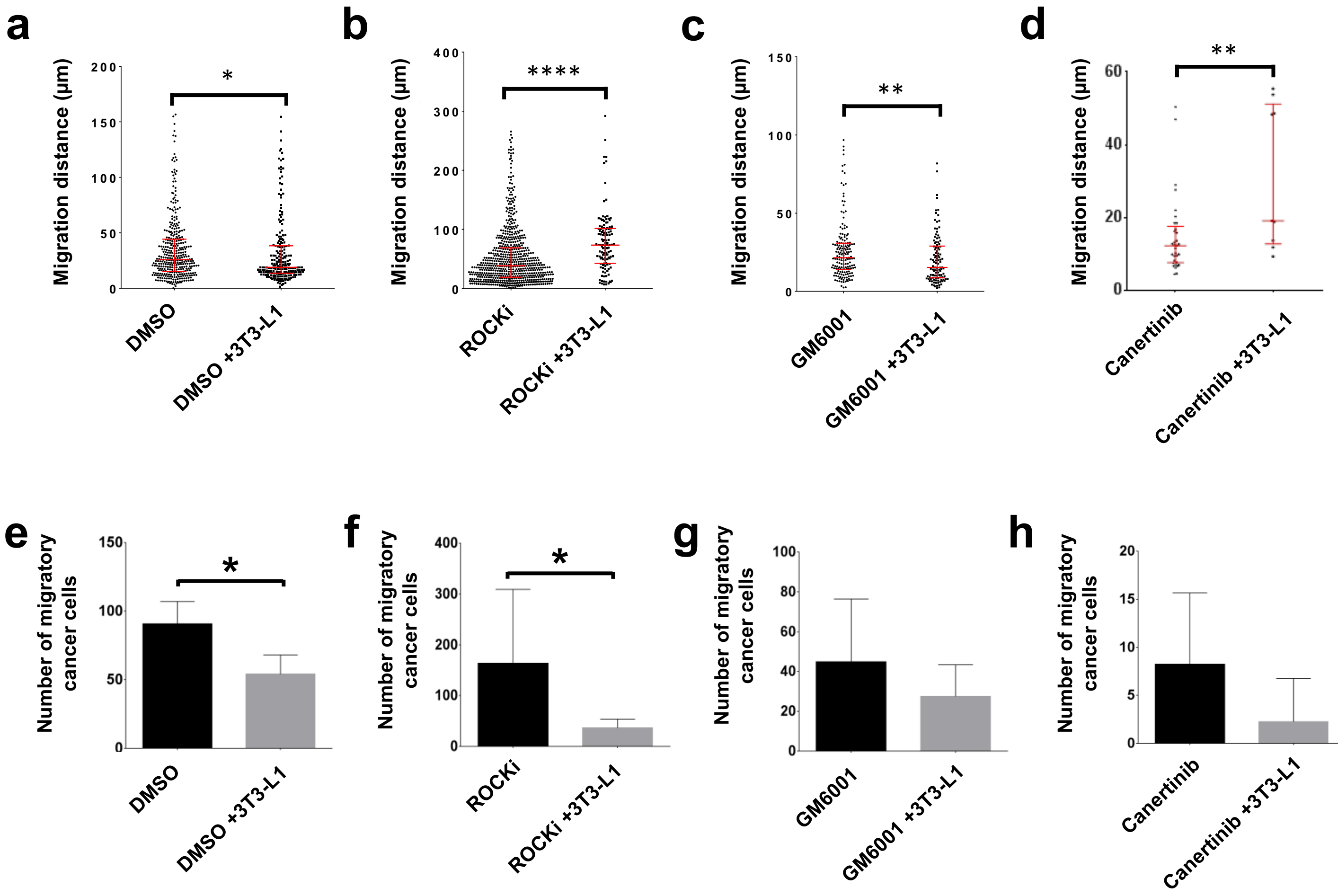

Supplementary Figure 3. Adipocyte influence on tumour cell migration distance and total migratory cell number (72 hours). Comparison of Wnt1 tumour cell migratory distance, from the anisotropic collagen scaffold nucleation point to within the scaffold, after 72 hours culture with DMSO (a), ROCKi (b), GM6001 (c) and Canertinib (d), all statistically compared with/without differentiated 3T3-L1 cells. The total number of migratory Wnt1 tumour cells within the scaffolds, after 72 hours culture with DMSO (e), ROCKi (f), GM6001 (g) and Canertinib (h), all statistically compared with/without differentiated 3T3-L1 cells. The non-parametric unpaired Mann-Whitney statistical test was applied to all cases. $*p<0.05$, $**p<0.01$, $****p<0.0001$

Supplementary Figure 4

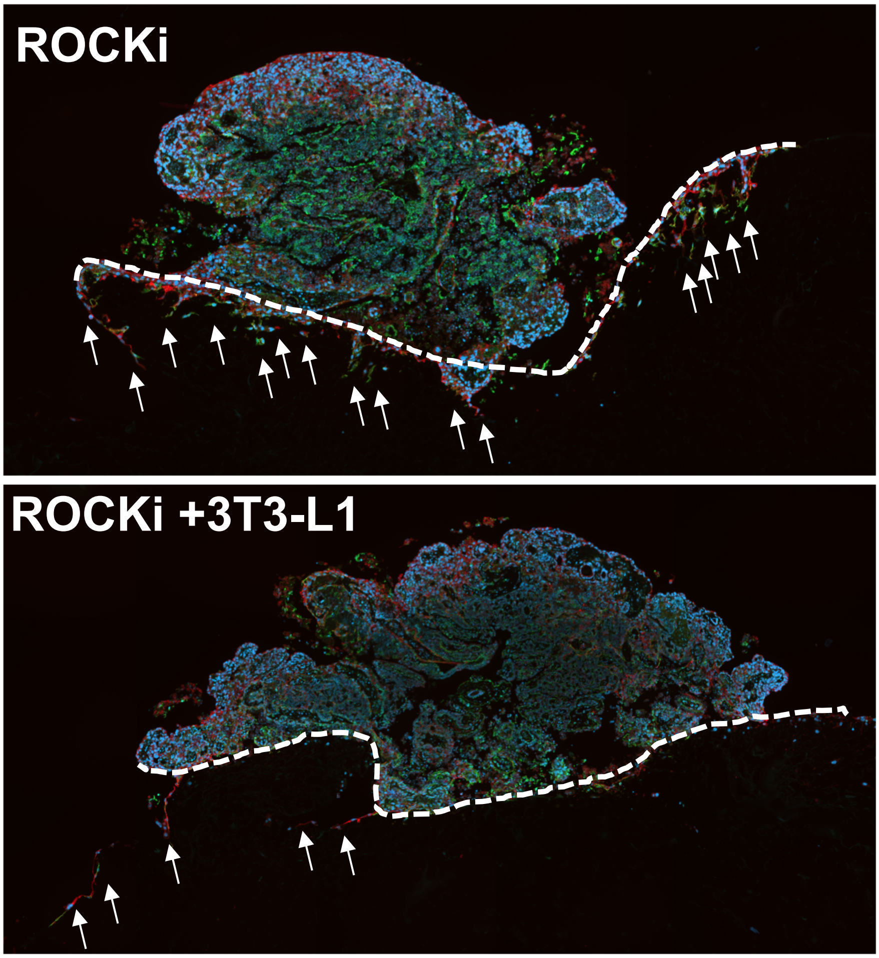

Supplementary Figure 4. Adipocyte co-culture increases migration distance and decreases total migratory cell number during treatment with ROCKi. Immunohistochemical analysis of Wnt1 tumour fragments in scaffolds, cultured for 72 hours with/without adipocytes (3T3-L1) and treated with ROCKi (Y-27632). Scaffold nucleation point is outlined (dotted line). Migratory cells are indicated with white arrows.

Supplementary Figure 5

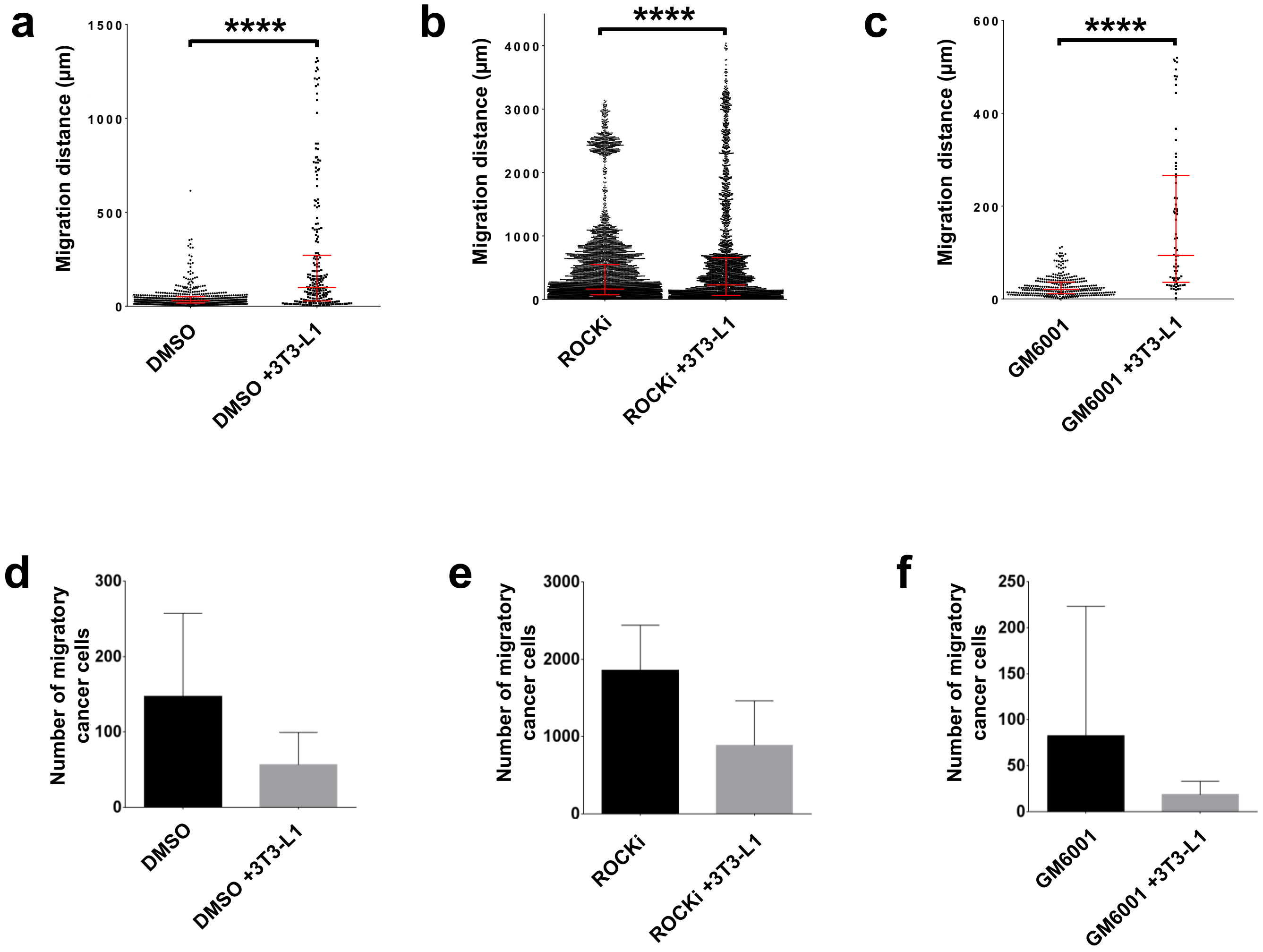

Supplementary Figure 5. Adipocyte influence on tumour cell migration distance and total migratory cell number (10 days). Comparison of Wnt1 tumour cell migratory distance, from the anisotropic collagen scaffold nucleation point to within the scaffold, after 10 days culture with DMSO (a), ROCKi (b) and GM6001 (c), all statistically compared with/without differentiated 3T3-L1 cells. The total number of migratory Wnt1 tumour cells within the scaffolds, after 72 hours culture with DMSO (e), ROCKi (f) and GM6001 (g), all statistically compared with/without differentiated 3T3-L1 cells. The non-parametric unpaired Mann-Whitney statistical test was applied to all cases. ****$p<0.0001$

Supplementary Figure 6

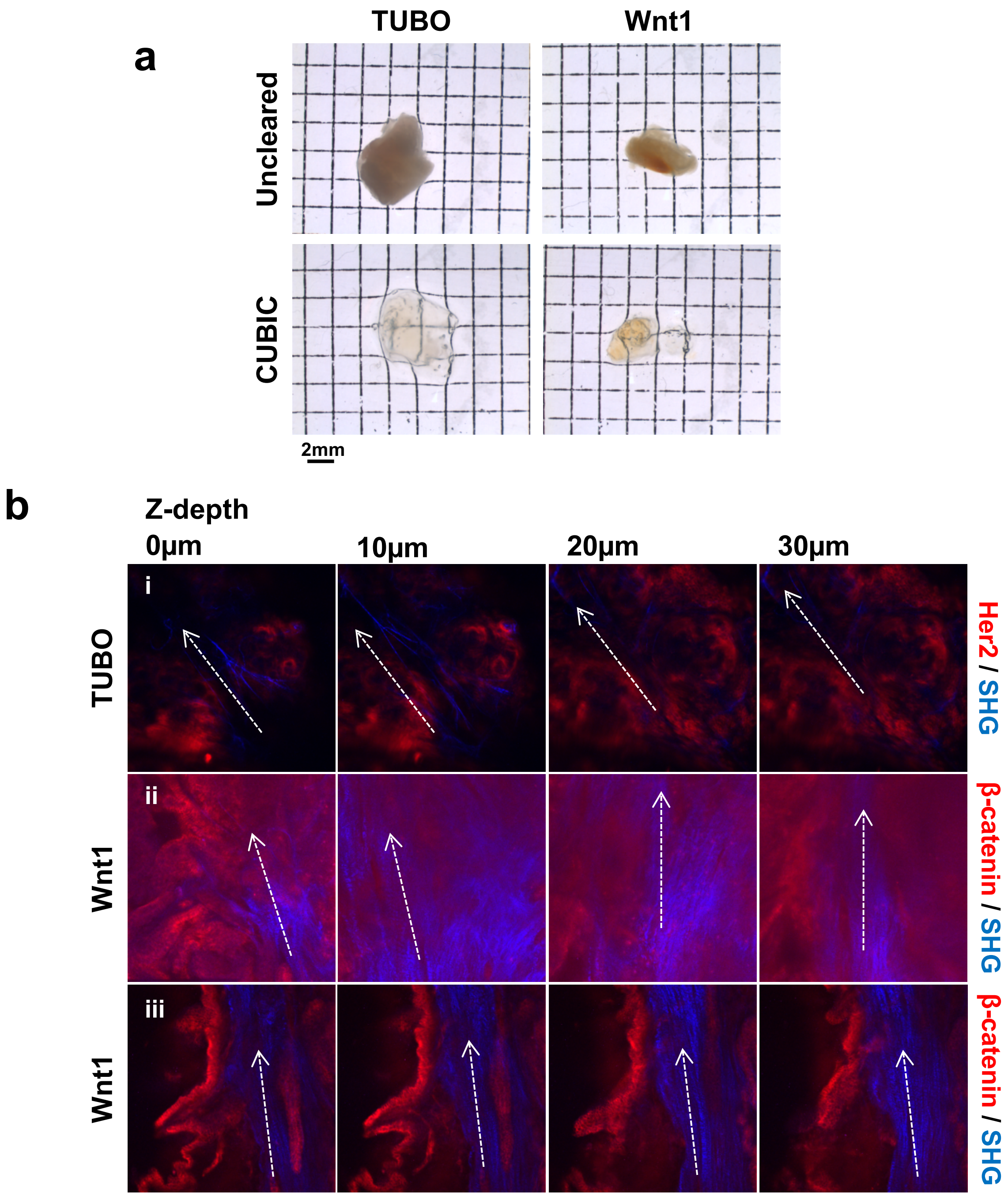

Supplementary Figure 6. CUBIC clearing, 2pf and SHG of tumours. (**a**) Transmission stereoscopic images of uncleared and CUBIC optically cleared TUBO (Her2-neu overexpressing) and Wnt1 tumours from a top down view (**b**) Whole mount CUBIC cleared tumours immunostained for Her2 (i, red) and β-catenin (ii-iii, red) and imaged using 2-photon fluorescence microscopy. Collagen (blue) was imaged directly using second harmonic generation. Directional collagen surrounding tumour cells is marked with a white arrow. Z-stacks are displayed as individual z-slices showing increasing depth from left to right.
